# Fascin Activates β-Catenin Signaling and Promotes Breast Cancer Stem Cell Function Mainly Through Focal Adhesion Kinase (FAK): Relation With Disease Progression

**DOI:** 10.3389/fonc.2020.00440

**Published:** 2020-04-21

**Authors:** Rayanah Barnawi, Samiyah Al-Khaldi, Tala Bakheet, Mohannad Fallatah, Ayodele Alaiya, Hazem Ghebeh, Monther Al-Alwan

**Affiliations:** ^1^Stem Cell and Tissue Re-Engineering Program, King Faisal Specialist Hospital and Research Centre, Riyadh, Saudi Arabia; ^2^National Center for Stem Cells, Life Science and Environment Research Institute, King Abdulaziz City for Sciences and Technology, Riyadh, Saudi Arabia; ^3^Molecular Biomedicine Program, King Faisal Specialist Hospital and Research Centre, Riyadh, Saudi Arabia; ^4^Collage of Medicine, Al-Faisal University, Riyadh, Saudi Arabia

**Keywords:** breast cancer, fascin, cancer stem cell, FAK, β-catenin

## Abstract

Cancer stem cells (CSCs), a rare population of tumor cells with high self-renewability potential, have gained increasing attention due to their contribution to chemoresistance and metastasis. We have previously demonstrated a critical role for the actin-bundling protein (fascin) in mediating breast cancer chemoresistance through activation of focal adhesion kinase (FAK). The latter is known to trigger the β-catenin signaling pathway. Whether fascin activation of FAK would ultimately trigger β-catenin signaling pathway has not been elucidated. Here, we assessed the effect of fascin manipulation in breast cancer cells on triggering β-catenin downstream targets and its dependence on FAK. Gain and loss of fascin expression showed its direct effect on the constitutive expression of β-catenin downstream targets and enhancement of self-renewability. In addition, fascin was essential for glycogen synthase kinase 3β inhibitor–mediated inducible expression and function of the β-catenin downstream targets. Importantly, fascin-mediated constitutive and inducible expression of β-catenin downstream targets, as well as its subsequent effect on CSC function, was at least partially FAK dependent. To assess the clinical relevance of the *in vitro* findings, we evaluated the consequence of fascin, FAK, and β-catenin downstream target coexpression on the outcome of breast cancer patient survival. Patients with coexpression of fascin^high^ and FAK^high^ or high β-catenin downstream targets showed the worst survival outcome, whereas in fascin^low^, patient coexpression of FAK^high^ or high β-catenin targets had less significant effect on the survival. Altogether, our data demonstrated the critical role of fascin-mediated β-catenin activation and its dependence on intact FAK signaling to promote breast CSC function. These findings suggest that targeting of fascin–FAK-β-catenin axis may provide a novel therapeutic approach for eradication of breast cancer from the root.

## Introduction

Recent years have witnessed increased overall survival of breast cancer patients that could be attributed to early detection and the substantial progress in developing more effective therapies. Nonetheless, tumor-related mortality remained high largely due to the presence of residual cancer cells, also called cancer stem cells (CSCs), which are chemoresistant and metastatic (1, 2). Fascin is an actin-bundling protein that is induced in many neoplasms including breast cancer (3), and we have previously showed that it enhances metastasis (4) and confers resistance to chemotherapy (5) through direct regulation of CSC population (6). Most recently, we have demonstrated that fascin enhances β1 integrin expression, a critical step for activation of the focal adhesion kinase (FAK) and subsequent regulation of CSC function (7). Other studies showed that FAK expression, which is often enhanced in breast cancer (8), is required for nuclear translocation of β-catenin and transcriptional activation of the β-catenin target genes (9). Whether fascin activation of FAK regulates breast CSCs through β-catenin signaling has not been elucidated.

β-Catenin signaling cascade is initiated when cytoplasmic β-catenin is stabilized and translocated to the nucleus where it interacts with members of the T-cell factor (TCF)/Lef transcription factors and subsequently activates expression of the target genes. Mounting evidences have shown a critical role for β-catenin signaling in enhancing fascin expression and increasing cancer cell migration in multiple types of cancer. Indeed, β-catenin/TCF was reported to bind fascin promoter and increase its expression in colon cancer (10, 11), which in turn enhances cell migration. Furthermore, activation of β-catenin signaling was found to promote adamantinomatous cancer cell migration by regulating fascin, a known target gene of β-catenin/TCF signaling (12). Migration of airway epithelial cells was found to be enhanced by IL-6 signaling, which upregulates fascin expression and induces β-catenin signaling (13). Treatment of breast cancer cells with 12-O-tetradecanoylphorbol 13-acetate activates the STAT3α and β-catenin signaling pathways, which promotes fascin gene transcription and augments cell migration (14). It is important to note that while fascin is a target of the β-catenin signaling pathway, it was also reported to activate Wnt/β-catenin signaling to promote epithelial-to-mesenchymal transition (EMT) of colon cancer cells (15). Moreover, expression of matrix metalloprotease 2 (MMP2) and MMP9, which we have previously reported to be controlled by fascin in breast cancer cells (4), has been described as downstream targets of the β-catenin signaling pathway (16).

Here, we assessed the impact of fascin gain and loss of function on β-catenin expression and its subsequent effect on breast CSC function. Our results demonstrated that fascin directly enhances the constitutive and inducible expression of β-catenin downstream targets in a FAK-dependent manner. Furthermore, fascin-mediated activation of β-catenin signaling via FAK was critical for promoting self-renewability of CSCs. Importantly, gene expression analysis in breast cancer patients demonstrated shorter survival when fascin^high^ was coexpressed with high FAK or high β-catenin downstream targets, demonstrating a clinical relevance of our *in vitro* data. Altogether, the data presented in this study demonstrated that fascin–FAK-β-catenin axis plays a critical role in promoting breast CSC function, and the interference with this axis may thus provide an attractive approach for therapeutic targeting of breast cancer from the root.

## Materials and Methods

### Cell Culture

The MDA-MB-231 (HTB-26) and T-47D (HTB-133) breast cancer cell lines were purchased from ATCC (Manassas, VA, USA) and were maintained in culture as previously described (7). Cells were routinely screened for mycoplasma using a polymerase chain reaction (PCR)–based kit (iNtRON, Sangdaewon-Dong, Jungwon-Gu, Seongnam-Si, Gyeonggi-do, Korea).

### Stable Transfection

Fascin stable knockdown (fascin^−^) and control (fascin^+^) were established in the naturally fascin-positive MDA-MB-231 cells using fascin and scrambled ShRNA, respectively, as previously described (4). Fascin was also ectopically expressed in the naturally fascin-negative T-47D cells or rescued in the fascin-knockdown (fascin^−^) MDA-MB-231 cells using fascin ORF (FORF) or control ORF as previously described (7). The knockdown and expression of fascin were routinely confirmed using Western blot.

### Reagents

The anti–fascin, β-catenin, TCF3, c-Myc, and GAPDH antibodies that were used in the Western blots were from Cell Signaling (Danvers, MA, USA). The anti–cyclin D1 antibody was from Abcam (Cambridge, MA, USA).

The focal adhesion kinase inhibitor (FAKi) and the glycogen synthase kinase 3β inhibitor (GSK-3βi) were obtained from Sigma (St. Louis, MO, USA), and the stock solution was diluted to desired concentrations in culture medium. Whenever applicable, cells were pretreated for 24 h with either 2 μM of FAKi or 5 μM of GSK-3βi or both, harvested, and added to the respective assay.

### Quantitative Real-Time PCR

First-strand complementary DNA (cDNA) was synthesized from ~2 μg of extracted total RNA using High Capacity RNA-to-cDNA Kit (Applied Biosystems, Paisley, UK). TaqMan fluorogenic probes for the specific gene were from Applied Biosystems (Thermo Fisher Scientific, Waltham, MA, USA) and all quantitative reverse transcriptase–PCR reactions were performed using Applied Biosystems 7500 Fast detection system. The relative mRNA expression was normalized against the housekeeping gene (GAPDH) and analyzed using 2^−ΔΔCT^ equation as previously described (4). Quantitative Real-Time PCR data were presented as mean of triplicates ± SD of three independent experiments.

### Western Blot

Cells lysates were prepared, and proteins were quantified as previously described (4). An optimized concentration of protein samples was loaded and transferred on iBlot premade PVDF membrane using dry transfer system (Bay Cities Tool & Supply Inc., Newark, CA, USA). Membranes were then blocked, washed, and incubated with the desired concentration of primary antibody for overnight before incubation with a horseradish peroxidase–conjugated secondary antibody for 45 min. The bounded antibodies were detected using chemiluminescence Super Signal System (Thermo Fisher Scientific) and were captured on ImageQuant LAS4010 Biomolecular Imager (GE Healthcare, Pittsburgh, PA, USA). Imager bands were quantified and analyzed using the QuantityOne software by Bio-Rad Laboratories, Inc (Hercules, CA, USA).

### Tumorsphere and Colony-Forming Assays

The tumorsphere assay was performed as previously described (6). Cells were seeded in a special medium, which was previously described by Dontu et al. (17), at 500 cells per well in 96-well ultralow attachment plates (Corning). Primary tumorspheres were counted before they were collected and dissociated to generate single cells for secondary tumorspheres. Tumorspheres (cutoff size ≥50 μm) were counted using EVOS digital inverted microscope by Thermo Fisher Scientific (Waltham, MA, USA).

The colony-forming assay was performed in 6-well plates (Corning) as previously described (6). MDA-MB-231 and T-47D cells were seeded at 100 and 2,500 cells/well in 3 mL of complete DMEM media, respectively. After 10 to 12 days in culture, media was removed, and the wells were washed, fixed, and stained with crystal violet to visualize and count colonies (blue dots) as previously described (6).

### Migration and Invasion Assays

Migration and invasion assays were performed using CIM-plate 16 on xCELLigence Real Time Cell Analysis instrument, from ACEA Biosciences Inc. (San Diego, CA, USA), which allows quantitative kinetic measurement of cell movement from the upper chamber to the lower chamber. Cells were cultured for 24 h until they reach 60–80% confluence followed by 6-h serum starvation [1% fetal bovine serum (FBS)] before they were harvested and adjusted at 30 × 10^3^/100 μL in serum-free media. For migration and invasion, 160 μL of chemotaxis (10% FBS) was added to the lower chamber, and cells (100 μL) were added to the upper chamber. For invasion, 25 μL of Matrigel was used to coat the upper chamber for 4 h in the 37°C incubator prior to the addition of cells. The cells were allowed to settle for 30 min at room temperature before loading the plate into the instrument, and measurements were taken at 15-min intervals for 24 h.

Data acquisition was stopped after 24 h of measurement, and the average migration or invasion of all the replicates was calculated using the xCELLigence software. Migration or invasion signal was considered positive if it has a mean Cell Index of ≥0.1. Rate of cell migration and invasion was assessed by increases in the slope (1/h) of the curve during 24 h, and a graph displaying change in the slope between the different groups was generated.

### Survival Analysis of Human Breast Tumor Microarray Datasets

The effect of a particular gene or multiple genes on breast cancer survival was assessed using the Kaplan–Meier plotter portal website (https://kmplot.com/analysis/) (18). The database covers more than 54,000 gene expression in 6,234 breast cancer patients, based on data extracted from the European Genome-Phenome Archive and the Gene Expression Omnibus repositories. All survival analysis data were downloaded in accordance with Kaplan–Meier plotter portal Access Policies. The different genes in the tumor samples were categorized into high and low expression, and the survival curves of samples with high and low gene expression were compared by log-rank test.

### Statistical Analysis

The analysis was performed using two-tailed paired Student *t*-test between control and treated samples. Data were presented in mean or replicates ± SD. Any *p*-value of <0.05 was considered statistically significant.

## Results

### Fascin Enhances the Expression of β-Catenin and Its Downstream Targets

**Figure 1:**
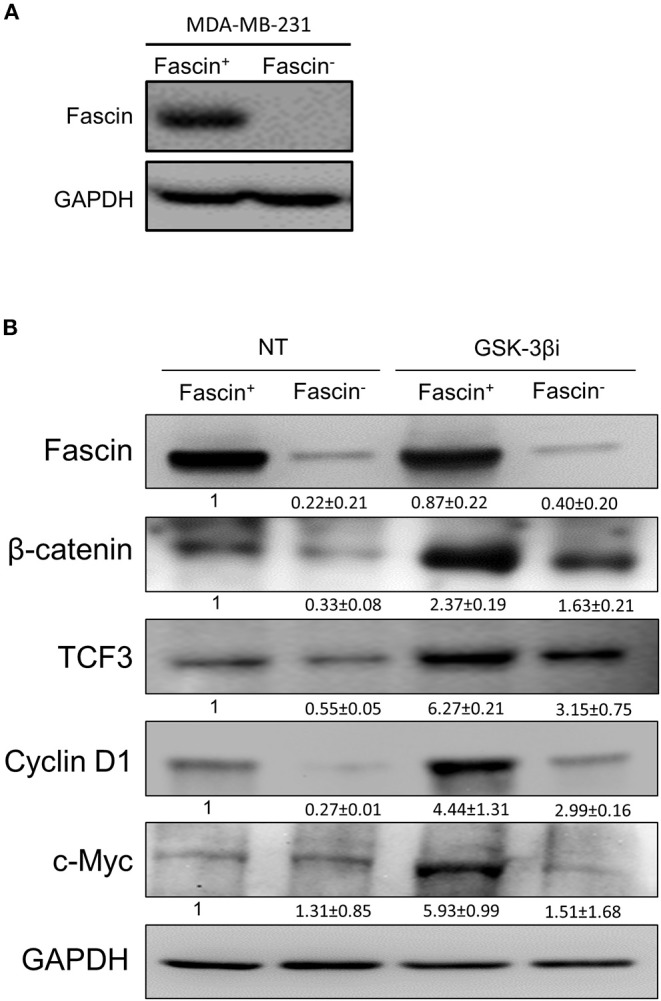
**(A,B)** Fascin knockdown reduces the expression of β-catenin and its downstream targets in breast cancer cells. **(A)** Western blot image showing fascin expression after its knockdown (fascin^−^) in MDA-MB-231 cells. **(B)** Western blot images showing the expression levels of fascin, β-catenin, TCF3, cyclin D1, and c-Myc following fascin knockdown in MDA-MB-231 cells before and after treatment with GSK-3βi. The expression of each protein was normalized on GAPDH, and the numbers shown below the images indicate the mean fold changes (of three independent experiments ± SD) in fascin^−^ in reference to fascin^+^ group or in GSK-3βi–treated fascin^+^ and fascin^−^ cells in reference to their respective untreated counterparts.

**Figure 2:**
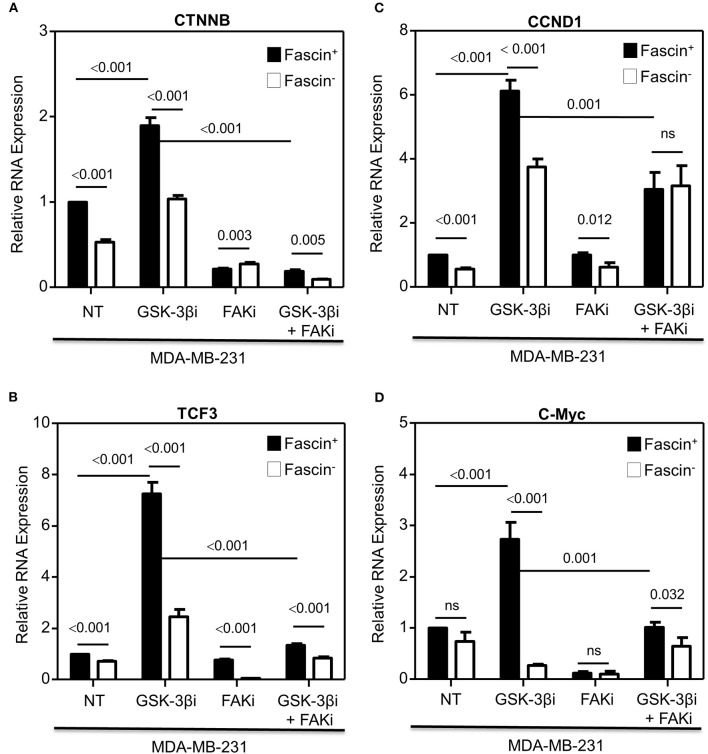
**(A–D)** Fascin activation of β-catenin downstream target genes is FAK-dependent. Bar graph showing relative RNA expression of CTNNB **(A)**, TCF3 **(B)**, CCND1 **(C)**, and c-Myc **(D)** following fascin knockdown in MDA-MB-231 cells before and after treatment with GSK-3βi ± FAKi. Results showing the mean of triplicates ± SD of three independent experiments, and each gene is normalized to the expression levels of untreated fascin^+^ cells.

Our previous work on breast cancer cells revealed a critical role for fascin in conferring chemoresistance via activation of FAK (5), a molecular adaptor that binds β1 integrin (19) and fascin (20) in breast cancer cells. In a subsequent study, we demonstrated that fascin expression in breast cancer cells induces β1 integrin to sustain FAK activation (7), which is also required for nuclear translocation of β-catenin and transcriptional activation of the β-catenin target genes (9). Whether FAK activates β-catenin signaling pathway in breast cancer and whether it is fascin-dependent have not been investigated. To this end, we have first tested whether fascin affects β-catenin protein expression. Knockdown of fascin (fascin^−^) in basal-like triple-negative MDA-MB-231 breast cancer cells (Figure 1A), which are naturally fascin positive (fascin^+^) (4), significantly (*p* < 0.05) reduced the protein expression of β-catenin, as well as TCF3 and cyclin D1, known β-catenin targets (Figure 1B). We then triggered β-catenin signaling pathway, through treatment with GSK-3βi, to assess the dependence of β-catenin activation on fascin expression. Treatment with GSK-3βi induces the expression of β-catenin, TCF3, cyclin D1, and c-Myc in fascin^+^ MDA-MB-231 cells at significantly (*p* < 0.05) higher levels as compared to their fascin^−^ counterparts (Figure 1B). To validate the effect of fascin expression on β-catenin signaling, we assessed the RNA expression level of standard β-catenin downstream targets following fascin knockdown. Constitutive gene expression of β-catenin (CTNNB) and its downstream targets TCF3 and cyclin D1 (CCND1) were significantly reduced in fascin^−^ MDA-MB-231 as compared to their fascin^+^ counterparts (Figures 2A–C). Consistent with the positive effect of fascin on β-catenin signaling, treatment with GSK-3βi significantly (*p* < 0.05) increased the inducible expression of CTNNB, TCF3, CCND1, and c-Myc in fascin^+^ MDA-MB-231 cells more than in their fascin^−^ counterparts (Figures 2A–D). We have previously established that fascin is critical for activation of FAK (7), which has been reported to trigger β-catenin signaling pathway (21). Thus, we have used FAKi to test if fascin-mediated β-catenin activation is FAK-dependent. Our fascin-mediated induction of CTNNB and its downstream targets through treatment with GSK-3βi was significantly reduced when FAKi was present (Figures 2B–D), demonstrating the dependence of fascin-mediated β-catenin activation on FAK.

To confirm that the effect on β-catenin downstream targets was due to fascin knockdown and not the results of fascin ShRNA off-target effect, fascin expression was restored using FORF in the fascin^−^ MDA-MB-231 cells (Supplementary Figure 1A). When compared to fascin^−^ MDA-MB-231 that was transfected with scrambled ShRNA (NORF), fascin-rescued cells (FORF) showed significantly increased expression of TCF3, CCND1, and c-Myc, which were comparable to that of the fascin^+^ (NORF) group (Supplementary Figures 1B–D). Fascin rescued (FORF) in fascin^−^ MDA-MB-231 cells significantly induced the expression of TCF3, CCND1, and c-Myc after treatment with GSK-3βi as compared to their NORF transfected fascin^−^ MDA-MB-231 cells. Fascin-mediated induction of β-catenin downstream targets after fascin rescued (FORF) in fascin^−^ MDA-MB-231 cells was diminished when treated with FAKi. To further validate the dependence of β-catenin signaling on fascin expression, FORF was used to express fascin in T-47D (Supplementary Figure 2A), hormone receptor–positive luminal breast cancer cells that are naturally fascin-negative and were compared to their scrambled transfected (NORF) counterparts (4). The expression of TCF3, CCND1, and c-Myc after GSK-3βi treatment was significantly induced in fascin-expressing T-47D (FORF) as compared to their fascin-negative (NORF) counterparts (Supplementary Figures 2B–D). Importantly, fascin-mediated induction of β-catenin downstream targets was diminished when fascin-expressing T-47D (FORF) was treated with FAKi. Fascin loss of function/restoration in MDA-MB-231 and gain of function in T-47D clearly demonstrated its positive effect on β-catenin and its downstream targets in a FAK-dependent manner regardless of their hormone receptor status.

### Fascin-Mediated β-Catenin Signaling Promotes Cancer Stem Cell Function

**Figure 3:**
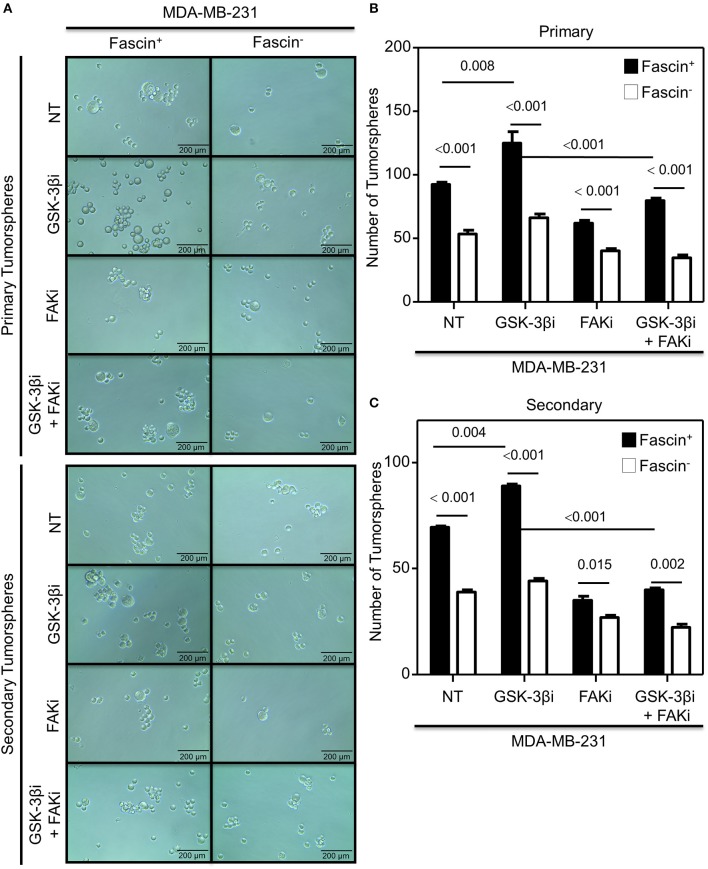
**(A–C)** Fascin activation of β-catenin signaling pathway enhances tumorsphere formation in a FAK-dependent manner. Bar graph showing the number of tumorspheres formed by fascin^+^ and fascin^−^ MDA-MB-231 cells before and after treatment with GSK-3βi ± FAKi. **(A)** Representative images showing primary (top) and secondary (bottom) tumorsphere formed by fascin^+^ and fascin^−^ MDA-MB-231 cells before and after treatment with GSK-3βi ± FAKi. Bar graphs showing primary **(B)** and secondary **(C)** tumorspheres as mean of five replicates ± SD of three independent experiments.

**Figure 4:**
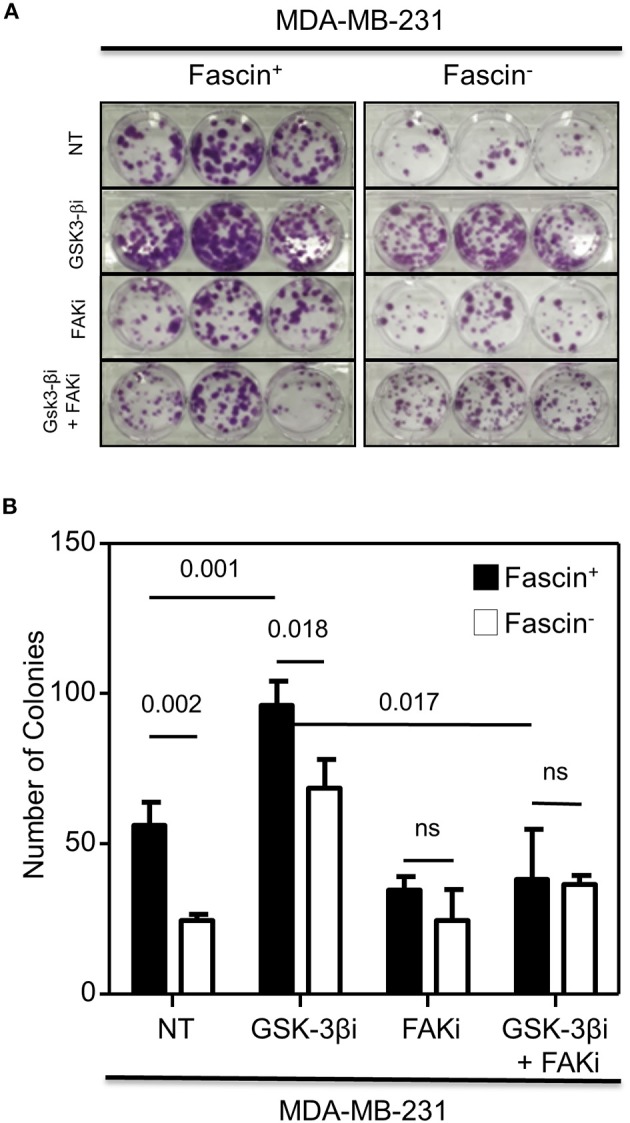
**(A,B)** Fascin activation of β-catenin signaling pathway promotes colony formation in a FAK-dependent manner. **(A)** Representative images showing colony formation by fascin^+^ and fascin^−^ MDA-MB-231 cells before and after treatment with GSK-3βi ± FAKi. **(B)** Bar graph showing the number (mean of triplicates ± SD) of colony formed by fascin^+^ and fascin^−^ MDA-MB-231 cells before and after treatment with GSK-3βi ± FAKi. The results are representative of three independent experiments.

**Figure 5:**
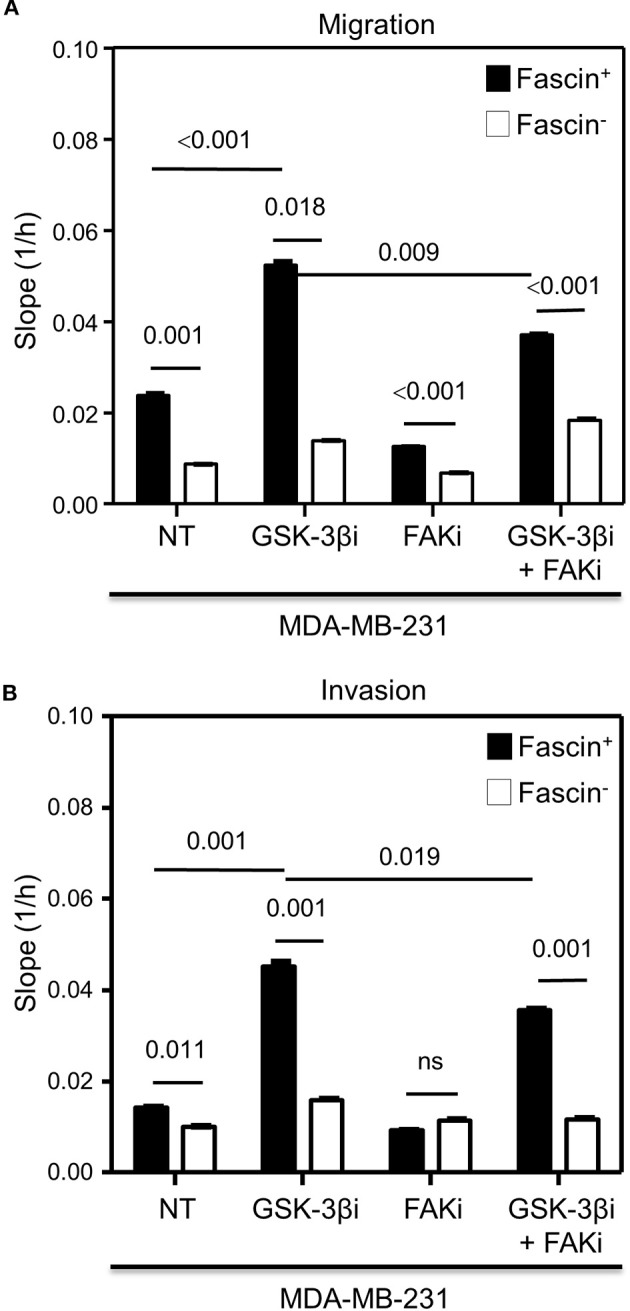
**(A,B)** Fascin activation of β-catenin signaling pathway increases migration and invasion in a FAK-dependent manner. The rate of migration and invasion was monitored in real time using the xCELLigence system. Bar graph showing the migration **(A)** or invasion **(B)** rate of fascin^+^ and fascin^−^ MDA-MB-231 cells before and after treatment with GSK-3βi ± FAKi. Migration and invasion rates were measured as increases in the slope (1/h) of the curve during 24 h and are mean of triplicates ± SD of three independent experiments.

The results above showed a critical role for fascin in regulating β-catenin signaling pathway in a FAK-dependent manner. We thus tested whether fascin induction of β-catenin downstream targets is reflected functionally on breast cancer self-renewability, as assessed by tumorsphere, colony formation, migration, and invasion assays. Consistent with the expression of higher levels of β-catenin, fascin^+^ MDA-MB-231 cells formed more primary and secondary tumorspheres as compared to their fascin^−^ counterparts (Figures 3A–C). Activation of the β-catenin signaling pathway through treatment with GSK-3βi significantly increases the number of primary and secondary tumorspheres formed by fascin^+^ MDA-MB-231 cells as compared to their fascin^−^ counterparts. Glycogen synthase kinase 3β inhibitor–mediated induction of tumorsphere formation by fascin^+^ MDA-MB-231 cells was lost in the presence of FAKi. Similar to what has been observed with the tumorsphere formation, fascin^+^ MDA-MB-231 cells formed more colonies as compared to their fascin^−^ counterparts (Figures 4A,B). Treatment with GSK-3βi significantly increases the number of colonies formed by fascin^+^ MDA-MB-231 cells as compared to their fascin^−^ counterparts. However, GSK-3βi failed to induce colony formation by fascin^+^ MDA-MB-231 cells in the presence of FAKi. We then assessed the effect of fascin-mediated activation of β-catenin signaling on migration and invasion, which have been shown to confer stemness properties (22). Fascin^+^ MDA-MB-231 cells showed higher level of migration and invasion as compared to their fascin^−^ counterparts (Figures 5A,B). Treatment with GSK-3βi increases migration and invasion of fascin^+^ MDA-MB-231 cells as compared to their fascin^−^ counterparts. Importantly, GSK-3βi–mediated induction of migration and invasion by fascin^+^ MDA-MB-231 cells was suppressed in the presence of FAKi, showing that fascin-mediated β-catenin activation leading to enhanced migration and invasion is at least partially dependent on intact FAK signaling.

To confirm the dependence of GSK-3βi–mediated induction of tumorsphere and colony formation in MDA-MB-231 on fascin expression, we assessed the effect of fascin restoration (FORF) on the function of fascin^−^ MDA-MB-231 cells as compared to their scrambled ORF (NORF) counterparts. Both tumorsphere (Supplementary Figure 3) and colony (Supplementary Figure 4) formation ability was rescued and induced upon treatment with the GSK-3βi when fascin expression was restored (FORF) in fascin^−^ MDA-MB-231 cells. However, treatment of fascin-restored MDA-MB-231 cells (FORF) with FAKi suppressed GSK-3βi–mediated induction of tumorsphere and colony formation. The dependence of GSK-3βi–mediated induction of tumorsphere and colony formation on fascin expression was also validated in the naturally fascin-negative T-47D breast cancer cells (NORF) and compared with their fascin-expressing counterparts (FORF). Glycogen synthase kinase 3β inhibitor failed to induce tumorsphere (Supplementary Figure 5) or colony (Supplementary Figure 6) formation in the fascin-negative T-47D cells (NORF). The effect of GSK-3βi in promoting tumorsphere and colony formation was observed when fascin was expressed in T-47D (FORF). Similar to what has been observed in the MDA-MB-231 cells, GSK-3βi–mediated induction of tumorsphere and colony formation that was dependent on fascin expression in T-47D (FORF) was lost in the presence of FAKi. Collectively, the gain, rescue, and loss of function data demonstrated that fascin-mediated activation of β-catenin signaling through FAK is essential for breast CSC function.

### Coexpression of Fascin and FAK or β-Catenin Downstream Targets Is Associated With Poor Survival

**Figure 6:**
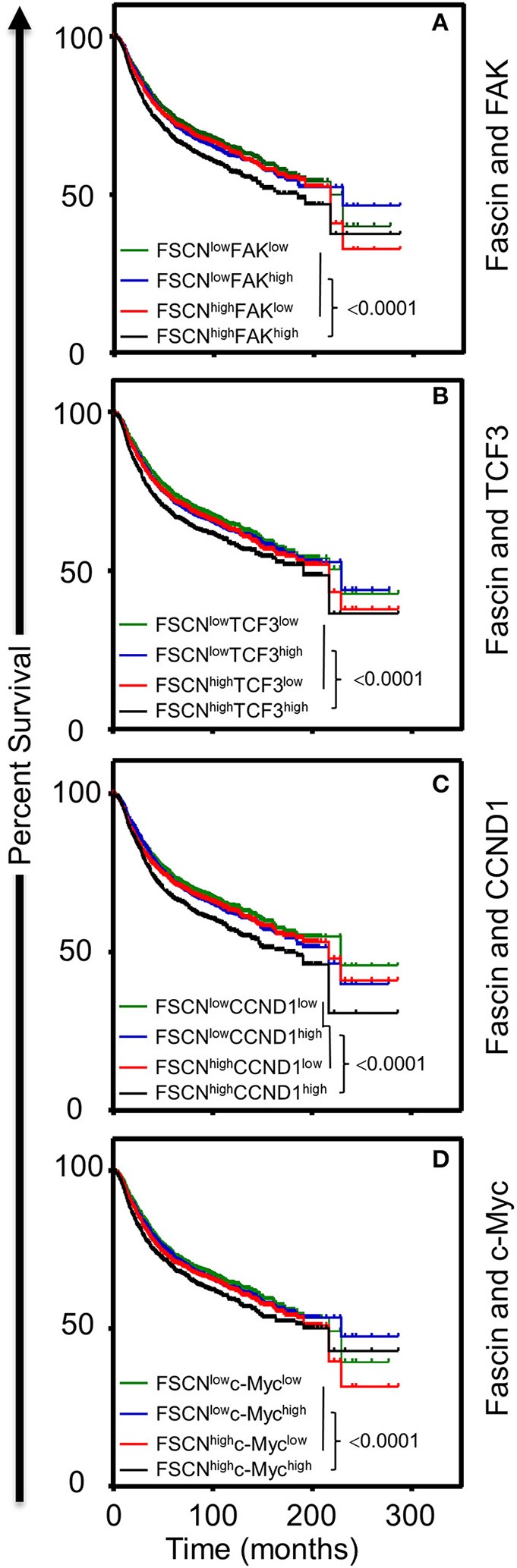
**(A–D)** Coexpression of fascin^high^ in conjunction with FAK^high^ and high β-catenin downstream targets correlates with poor breast cancer survival. Kaplan–Meier plot showing recurrence-free survival (RFS; *n* = 3,951) of TCGA breast cancer cohort for **(A)** fascin and FAK, **(B)** fascin and TCF3, **(C)** fascin and CCND1, **(D)** fascin and c-Myc. Patients were grouped into high or low based on the respective gene expression, and significance between expression groups was calculated using the log-rank test. *P*-values are indicated on each plot.

**Figure 7:**
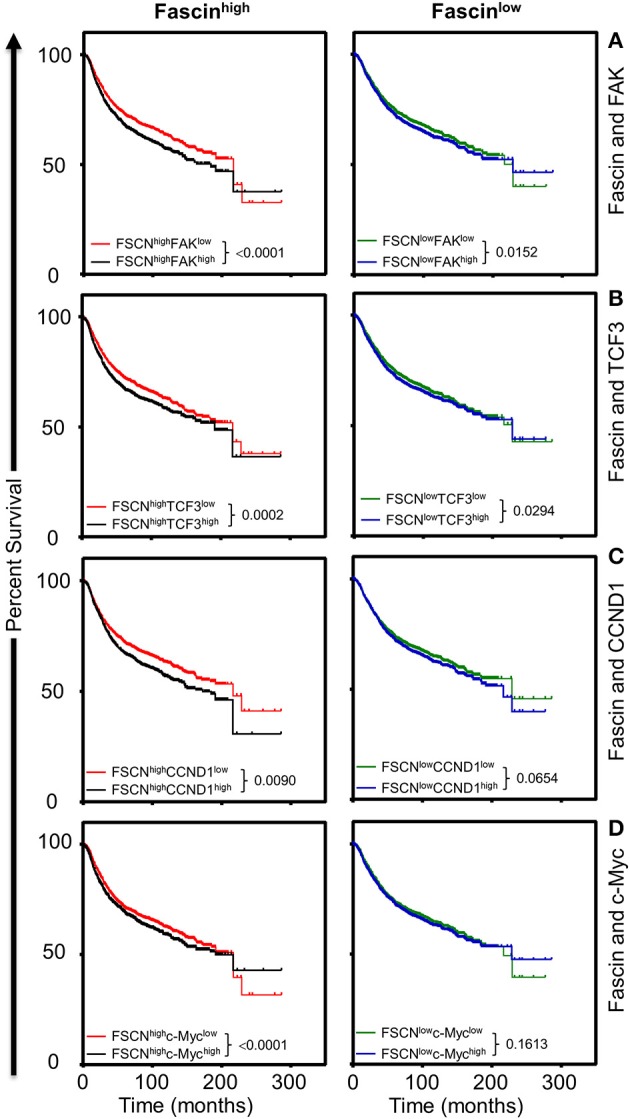
**(A–D)** The influence of FAK^high^ and high β-catenin downstream targets on poor survival is more significant in fascin^high^ breast cancer patients. Kaplan–Meier plot showing the significance of FAK and β-catenin downstream targets on RFS (*n* = 3,951) according to fascin expression (fascin^high^ and fascin^low^). Patients were grouped into fascin^high^ (left) and fascin^low^ (right), and the significance of the respective gene expression in each group was calculated using the log-rank test. *P*-values are indicated on each plot.

The *in vitro* results above showed that fascin-mediated β-catenin signaling was FAK-dependent and indicated that this process was critical for promoting breast CSC function. To understand the clinical relevance of these findings, we have used the publicly available Kaplan–Meier plotter portal (18) dataset to determine the impact of fascin coexpression with β-catenin transcriptional genes on breast cancer recurrence-free survival (RFS; *n* = 3,951). The survival analysis revealed that patients with fascin^high^/FAK^high^ expression have the worst RFS as compared with fascin^high^/FAK^low^, fascin^low^/FAK^high^ fascin^low^/FAK^low^-expressing patients (Figure 6A). Similarly, fascin^high^/TCF3^high^, fascin^high^/CCND1^high^, and fascin^high^/c-Myc^high^ patients showed the worst RFS as compared to the three other combinations of patients (Figures 6B–D). To determine whether FAK and β-catenin downstream targets effect on RFS was fascin dependent, we stratified the survival data according to the expression level of fascin (fascin^high^ and fascin^low^). The power of significance (*p*-value) of FAK^high^ effect on RFS was dramatically reduced from <0.0001 in fascin^high^ to 0.0152 in fascin^low^ patients (Figure 7A). Similarly, the power of significance of TCF3^high^ effect on RFS was dramatically reduced from 0.0002 in fascin^high^ to 0.0294 in fascin^low^ patients (Figure 7B). While CCND1^high^ expression showed significant (*p* = 0.0090) effect on worse RFS in fascin^high^ patients, this significance was lost (*p* = 0.0654) in fascin^low^ patients (Figure 7C). Similarly, c-Myc^high^ expression showed significant (*p* < 0.0001) effect on worse RFS in fascin^high^ patients; this significance was lost (*p* = 0.1613) in fascin^low^ patients (Figure 7D). The fact that the power of significance was either lost (CCND1^high^ or c-Myc^high^) or dramatically reduced (FAK^high^ or TCF3^high^) in fascin^low^ patients strongly suggests that the observed effect of FAK^high^ and high β-catenin targets on RFS in fascin^high^ patients was at least partially dependent on fascin expression. The poor RFS in patients that coexpress fascin^high^ with FAK^high^ or high β-catenin downstream targets demonstrated a clinical relevance to our *in vitro* findings. Collectively, these data suggest that fascin, FAK, and β-catenin downstream targets promote progression of breast cancer, and their levels of coexpression could serve as biomarkers for assessing the prognosis of the disease.

## Discussion

Growing evidence has shown that fascin is not only a target of the β-catenin signaling pathway, but it can also activate Wnt/β-catenin signaling to promote EMT (15), a feature of CSCs. We have previously demonstrated that fascin confers chemoresistance in breast cancer cells via activation of FAK (5), which is sustained through fascin induction of β1 integrin (7). Moreover, the enhanced FAK expression reported in breast cancer (8) was found to be required for β-catenin signaling and transcriptional activation of its target genes (9). In the present study, we have demonstrated a direct effect of fascin on the constitutive and inducible β-catenin activation in breast cancer cells. Importantly, fascin-mediated β-catenin activation promotes CSC function in a FAK-dependent manner. In line with our *in vitro* results, analysis of the publicly available survival dataset showed poor RFS in patients that coexpress fascin^high^ with FAK^high^ or high β-catenin downstream targets, demonstrating a functional relationship between these proteins that associate with worse patient survival outcome.

Increased expressions of fascin, β-catenin, and FAK were independently reported in breast cancer and were found to associate with enhanced self-renewability. In particular, the Wnt/β-catenin signaling pathway controls many cellular processes, but was also reported to be deregulated in many type of cancers including breast (23). Indeed, we have previously reported that fascin activates FAK (5, 7), and another study demonstrated fascin triggering of β-catenin signaling in cholangiocarcinoma (15). Moreover, FAK was reported to be critical for β-catenin signaling and transcriptional activation of its target genes (9). Through use of fascin loss and rescue in triple-negative basal-like and gain function approaches in hormone receptor–positive luminal breast cancer cells, this study investigated whether fascin activation of FAK would ultimately trigger β-catenin signaling. The data presented in this study demonstrated a direct effect of fascin on the constitutive and inducible β-catenin expression and the dependence of this activation process on intact FAK signaling. The fact that this phenomenon was observed in hormone receptor–negative basal-like (MDA-MB-231) and hormone receptor–positive luminal (T-47D) breast cancer cells strongly suggests that fascin/FAK activates β-catenin signaling pathway regardless of the hormone receptor status or breast cancer subtypes. This fascin-mediated β-catenin signaling was reflected on promoting CSC function in a FAK-dependent manner, providing a strong functional evidence for the interaction of these molecules in a cascade of events that fuel self-renewability. Our results of fascin-mediated β-catenin activation enhancing self-renewability and migration of breast CSCs are consistent with those of Jang et al. (24), where they demonstrated Wnt/β-catenin signaling to be critical for self-renewability and migration of breast CSCs. Taken together our previous study's finding that fascin confers chemoresistance through FAK activation (5) and the present findings that fascin-mediated β-catenin signaling enhances CSC function in FAK-dependent manner, one could envisage the crucial role of this axis in breast cancer prognosis and the therapeutic potential of its targeting. For instance, breast cancer–initiating cells were eliminated *in vitro* and *in vivo* when Wnt/β-catenin signaling was inhibited with small molecule antagonist (25), and β-catenin inhibition sensitized PIK3CA-mutant breast cancer to PI3K inhibitors (26). FAK targeting through miR-7 was also found to inhibit breast cancer metastasis and EMT (8). Chen et al. (27) demonstrated the ability of migrastatin, a small molecule that selectively target fascin, to block breast cancer metastasis to the lung. These findings provide compelling rational for the therapeutic targeting of fascin/FAK/β-catenin axis to induce breast cancer remission.

The poor survival that we observed in patients that express fascin^high^ and high β-catenin downstream targets is in line with the data reported by Shen et al. (28), where high β-catenin expression was shown to associate with poor overall and disease-specific survival in triple-negative breast cancers. Importantly, our data showed the worst survival outcome in patients coexpressing fascin^high^ with FAK^high^ or high β-catenin targets, whereas high expression of FAK or β-catenin targets has less significant effect on the survival of fascin^low^ patients. The survival data together with our *in vitro* results strongly suggest the dependence of β-catenin expression and function on fascin expression and the central role of FAK in this fascin-mediated β-catenin expression. The patient data reported in this study indicated poor survival when fascin^high^ was coexpressed with FAK^high^ or with high β-catenin downstream targets, strongly suggesting that their coexpression presents challenge for effective treatment of cancer. The demonstrated role of fascin in regulating breast cancer chemoresistance, metastasis, and CSC function makes it recognized as an attractive therapeutic target for the treatment of cancer. Our reported results provided insight into the role of fascin in mediating β-catenin activation through FAK and thus may give a new dimension to the field of breast cancer developmental therapeutics. Given that there are only limited studies that have targeted fascin through small molecules (27, 29) and based on the insight we gained from this study, one may consider targeting of FAK or β-catenin that appears to be main effectors of fascin. Galectin-3, a β-galactoside–binding protein that is upregulated in multiple types of cancers including breast, was found to promote β-catenin/Wnt signaling in tongue squamous cell carcinoma and breast cancer cells (30). Interestingly, it was demonstrated that galectin-3–mediated β-catenin expression through FAK activation promotes osteosarcoma cell migration, and chemoresistance (21). Kim et al. (11) suppressed the nuclear localization of β-catenin and its binding to the fascin promoter through silencing of galectin-3, providing another evidence for the therapeutic potential of targeting this axis.

The findings reported in this study of fascin-activating β-catenin signaling through FAK and the subsequent effect of this signaling cascade on CSC function present a feasible window for therapeutic targeting of fascin-FAK-β-catenin axis in order to effectively exterminate the core of cancer.

## Data Availability Statement

All datasets generated for this study are included in the article/Supplementary Material.

## Author Contributions

RB and SA-K performed the experiments and analyzed some of the data. TB performed all patient survival analysis using kmplot website. MF obtained fund for SA-K and provided materials support. AA edited the manuscript. HG performed some data interpretation and edited the manuscript. MA-A conceived and designed the experiments, analyzed the data, and wrote the paper.

## Conflict of Interest

The authors declare that the research was conducted in the absence of any commercial or financial relationships that could be construed as a potential conflict of interest.

## Supplementary Material

Supplementary Figure 1 **(A–D)** Rescue of fascin expression in the fascin^−^ MDA-MB-231 breast cancer cells restores activation of β-catenin downstream targets in a FAK-dependent manner. **(A)** Western blot image showing fascin expression in fascin^−^ MDA-MB-231 cells that were transfected with negative ORF (NORF) or fascin ORF (FORF). Bar graph showing relative RNA expression of TCF3 **(B)**, CCND1 **(C)**, and c-Myc **(D)** after fascin restoration (fascin^−^ with FORF) relative to fascin^−^ with NORF and fascin^+^ (fascin^+^ with NORF) groups in the presence or absence of GSK-3βi ± FAKi. Results showing the mean of triplicates ± SD of 3 independent experiments and each gene is normalized to the expression levels of untreated fascin^+^ cells (fascin^+^ with NORF).

Supplementary Figure 2 **(A–D)** Induction of fascin expression in the fascin-negative T-47D breast cancer cells increases activation of β-catenin downstream targets in a FAK-dependent manner. **(A)** Western blot image showing fascin expression in T-47D cells that were transfected with negative ORF (NORF) or fascin ORF (FORF). Bar graph showing relative RNA expression of TCF3 **(B)**, CCND1 **(C)**, and c-Myc **(D)** after fascin expression (FORF) in T-47D relative to NORF group in the presence or absence of GSK-3βi ± FAKi. Results showing the mean of triplicates ± SD of 3 independent experiments and each gene is normalized to the expression levels of untreated fascin-negative T-47D cells (NORF).

Supplementary Figure 3 **(A,B)** Rescue of fascin expression in the fascin^−^ MDA-MB-231 breast cancer cells restores their activation of β-catenin signaling pathway and enhances their tumorsphere formation ability in a FAK-dependent manner. Bar graph showing the number of tumorspheres formed after fascin restoration (fascin^−^ with FORF) relative to fascin^−^ with NORF and fascin^+^ (fascin^+^ with NORF) groups in the presence or absence of GSK-3βi ± FAKi. Primary **(A)** and secondary **(B)** tumorspheres are mean of 5 replicates ± SD of three independent experiments.

Supplementary Figure 4 Rescue of fascin expression in the fascin^−^ MDA-MB-231 breast cancer cells restores their activation of β-catenin signaling pathway and enhances their colony formation ability in a FAK-dependent manner. Colony formation was assessed after fascin restoration (fascin^−^ with FORF) relative to fascin^−^ with NORF and fascin^+^ (fascin^+^ with NORF) groups in the presence or absence of GSK-3βi ± FAKi. Bar graph showing the number (mean of triplicates ± SD) of colonies of three independent experiments.

Supplementary Figure 5 **(A,B)** Induction of fascin expression in the fascin-negative T-47D breast cancer cells increases their activation of β-catenin signaling pathway and enhances their tumorsphere formation ability in a FAK-dependent manner. Bar graph showing the number of tumorspheres formed after fascin expression (FORF) in T-47D relative to NORF group in the presence or absence of GSK-3βi ± FAKi. Primary **(A)** and secondary **(B)** tumorspheres are mean of 5 replicates ± SD of three independent experiments.

Supplementary Figure 6 Induction of fascin expression in the fascin-negative T-47D breast cancer cells increases their activation of β-catenin signaling pathway and enhances their colony formation ability in a FAK-dependent manner. Colony formation was assessed after fascin expression (FORF) in T-47D relative to NORF group in the presence or absence of GSK-3βi ± FAKi. Bar graph showing the number (mean of triplicates ± SD) of colonies of 3 independent experiments.
