# Supplementary figures and images for: Fascin Activates β-Catenin Signaling and Promotes Breast Cancer Stem Cell Function Mainly Through Focal Adhesion Kinase (FAK): Relation With Disease Progression
Source: Front Oncol. 2020 Apr 21;10:440. doi: 10.3389/fonc.2020.00440 (PMC7186340; doi:10.3389/fonc.2020.00440)

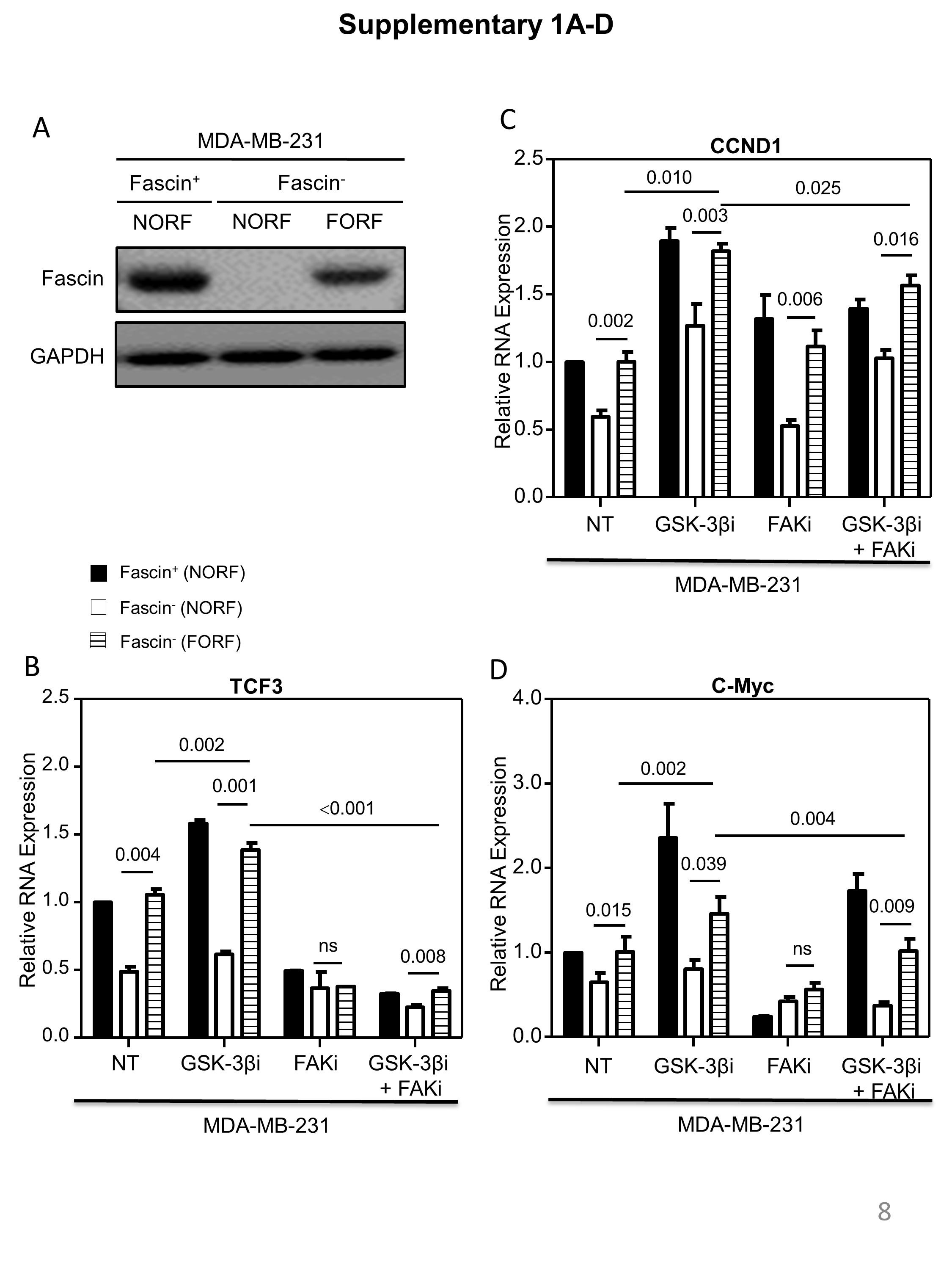

Supplement: Supplementary Figure 1 — (A–D) Rescue of fascin expression in the fascin− MDA-MB-231 breast cancer cells restores activation of β-catenin downstream targets in a FAK-dependent manner. (A) Western blot image showing fascin expression in fascin− MDA-MB-231 cells that were transfected with negative ORF (NORF) or fascin ORF (FORF). Bar graph showing relative RNA expression of TCF3 (B), CCND1 (C), and c-Myc (D) after fascin restoration (fascin− with FORF) relative to fascin− with NORF and fascin+ (fascin+ with NORF) groups in the presence or absence of GSK-3βi ± FAKi. Results showing the mean of triplicates ± SD of 3 independent experiments and each gene is normalized to the expression levels of untreated fascin+ cells (fascin+ with NORF). [file Image_1.TIF]

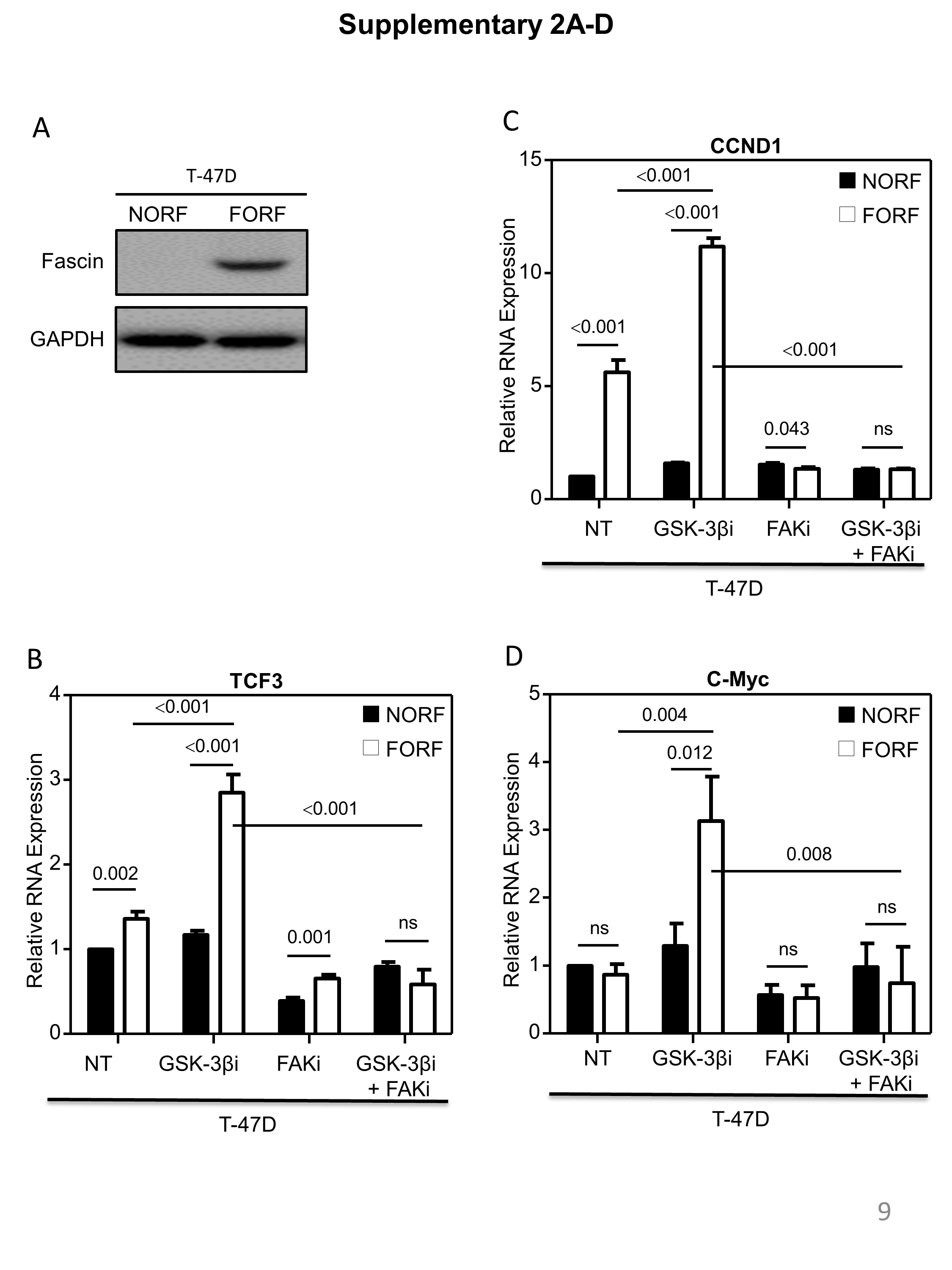

Supplement: Supplementary Figure 2 — (A–D) Induction of fascin expression in the fascin-negative T-47D breast cancer cells increases activation of β-catenin downstream targets in a FAK-dependent manner. (A) Western blot image showing fascin expression in T-47D cells that were transfected with negative ORF (NORF) or fascin ORF (FORF). Bar graph showing relative RNA expression of TCF3 (B), CCND1 (C), and c-Myc (D) after fascin expression (FORF) in T-47D relative to NORF group in the presence or absence of GSK-3βi ± FAKi. Results showing the mean of triplicates ± SD of 3 independent experiments and each gene is normalized to the expression levels of untreated fascin-negative T-47D cells (NORF). [file Image_2.TIF]

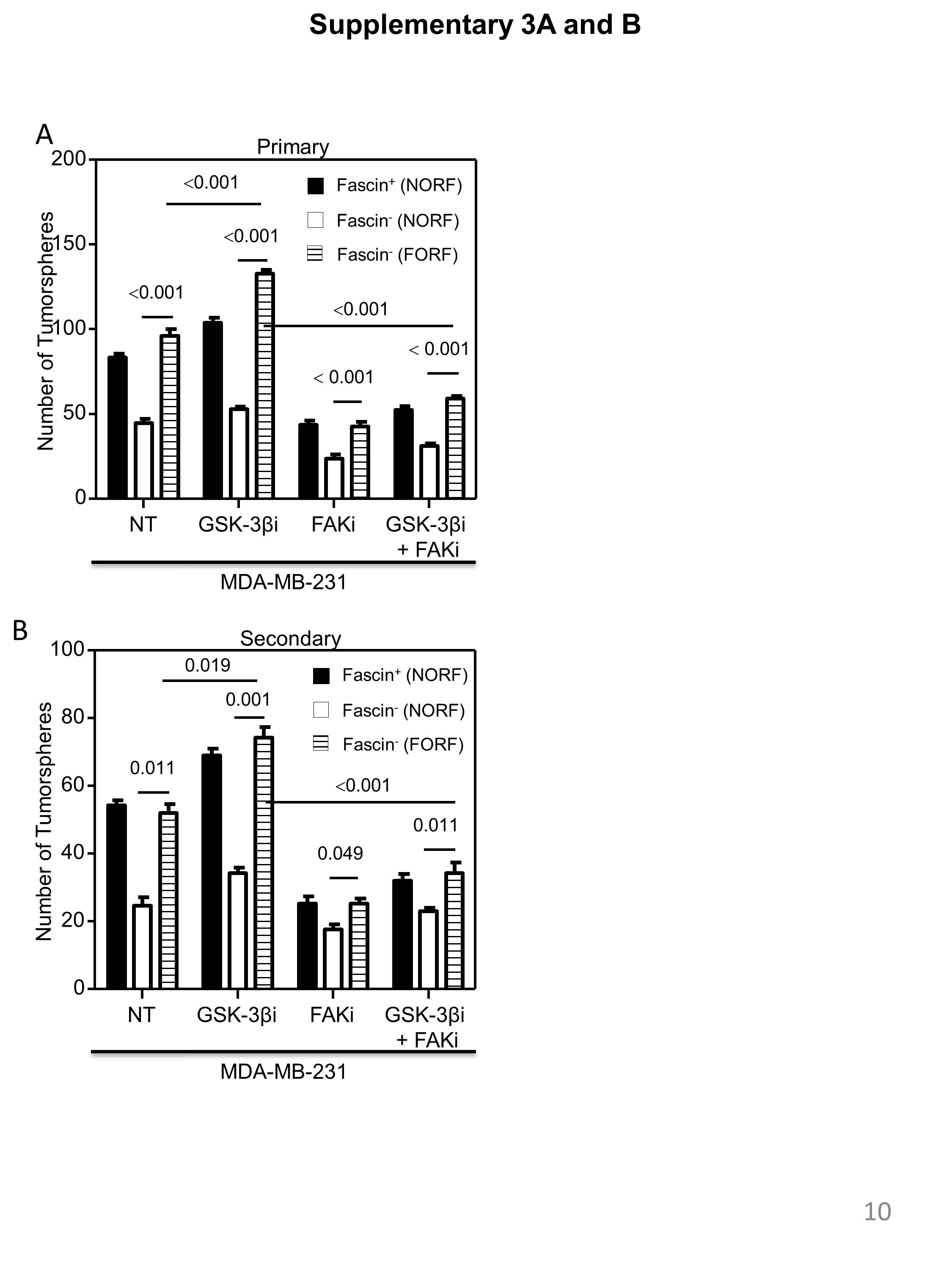

Supplement: Supplementary Figure 3 — (A,B) Rescue of fascin expression in the fascin− MDA-MB-231 breast cancer cells restores their activation of β-catenin signaling pathway and enhances their tumorsphere formation ability in a FAK-dependent manner. Bar graph showing the number of tumorspheres formed after fascin restoration (fascin− with FORF) relative to fascin− with NORF and fascin+ (fascin+ with NORF) groups in the presence or absence of GSK-3βi ± FAKi. Primary (A) and secondary (B) tumorspheres are mean of 5 replicates ± SD of three independent experiments. [file Image_3.TIF]

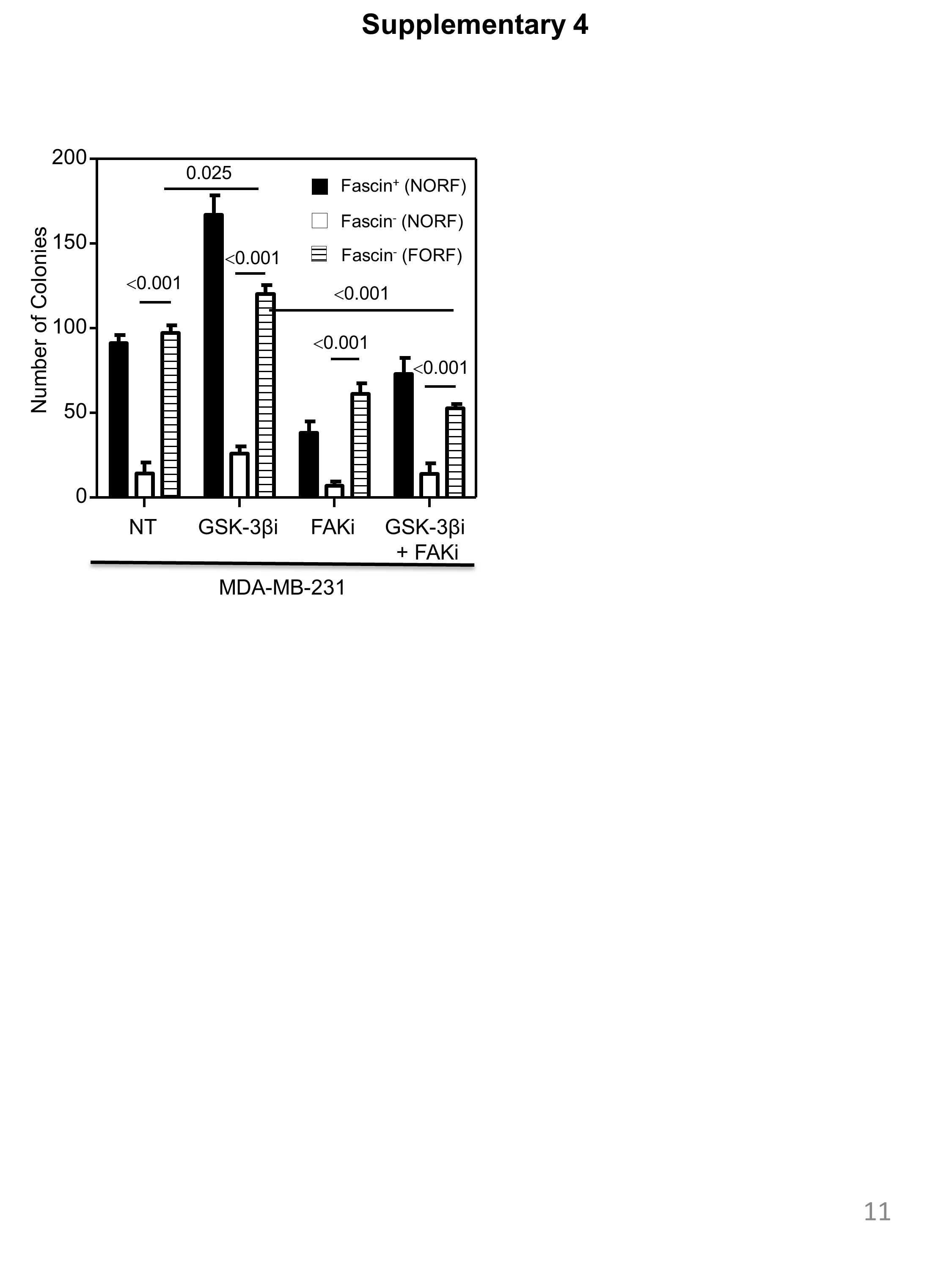

Supplement: Supplementary Figure 4 — Rescue of fascin expression in the fascin− MDA-MB-231 breast cancer cells restores their activation of β-catenin signaling pathway and enhances their colony formation ability in a FAK-dependent manner. Colony formation was assessed after fascin restoration (fascin− with FORF) relative to fascin− with NORF and fascin+ (fascin+ with NORF) groups in the presence or absence of GSK-3βi ± FAKi. Bar graph showing the number (mean of triplicates ± SD) of colonies of three independent experiments. [file Image_4.TIF]

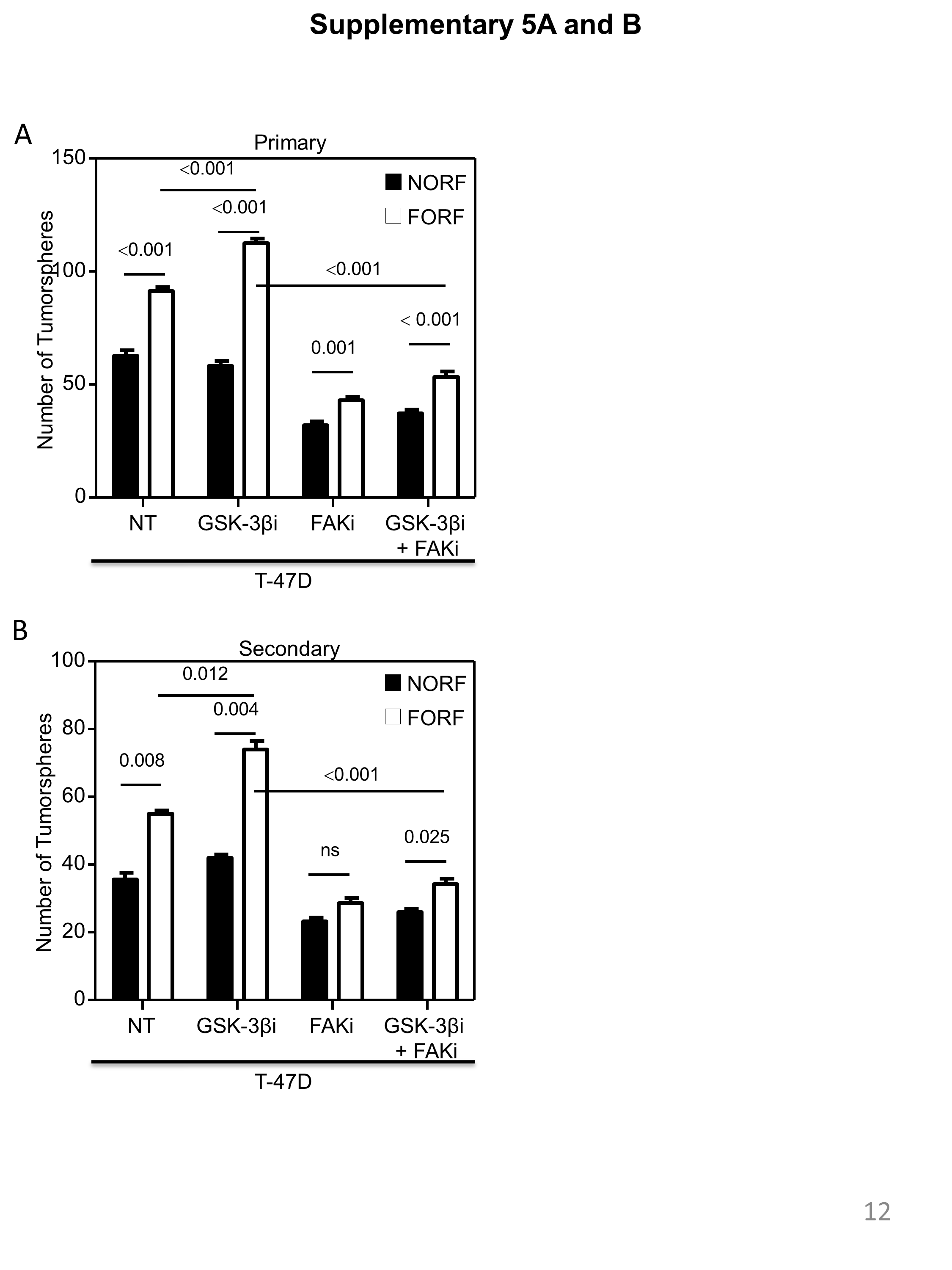

Supplement: Supplementary Figure 5 — (A,B) Induction of fascin expression in the fascin-negative T-47D breast cancer cells increases their activation of β-catenin signaling pathway and enhances their tumorsphere formation ability in a FAK-dependent manner. Bar graph showing the number of tumorspheres formed after fascin expression (FORF) in T-47D relative to NORF group in the presence or absence of GSK-3βi ± FAKi. Primary (A) and secondary (B) tumorspheres are mean of 5 replicates ± SD of three independent experiments. [file Image_5.TIF]

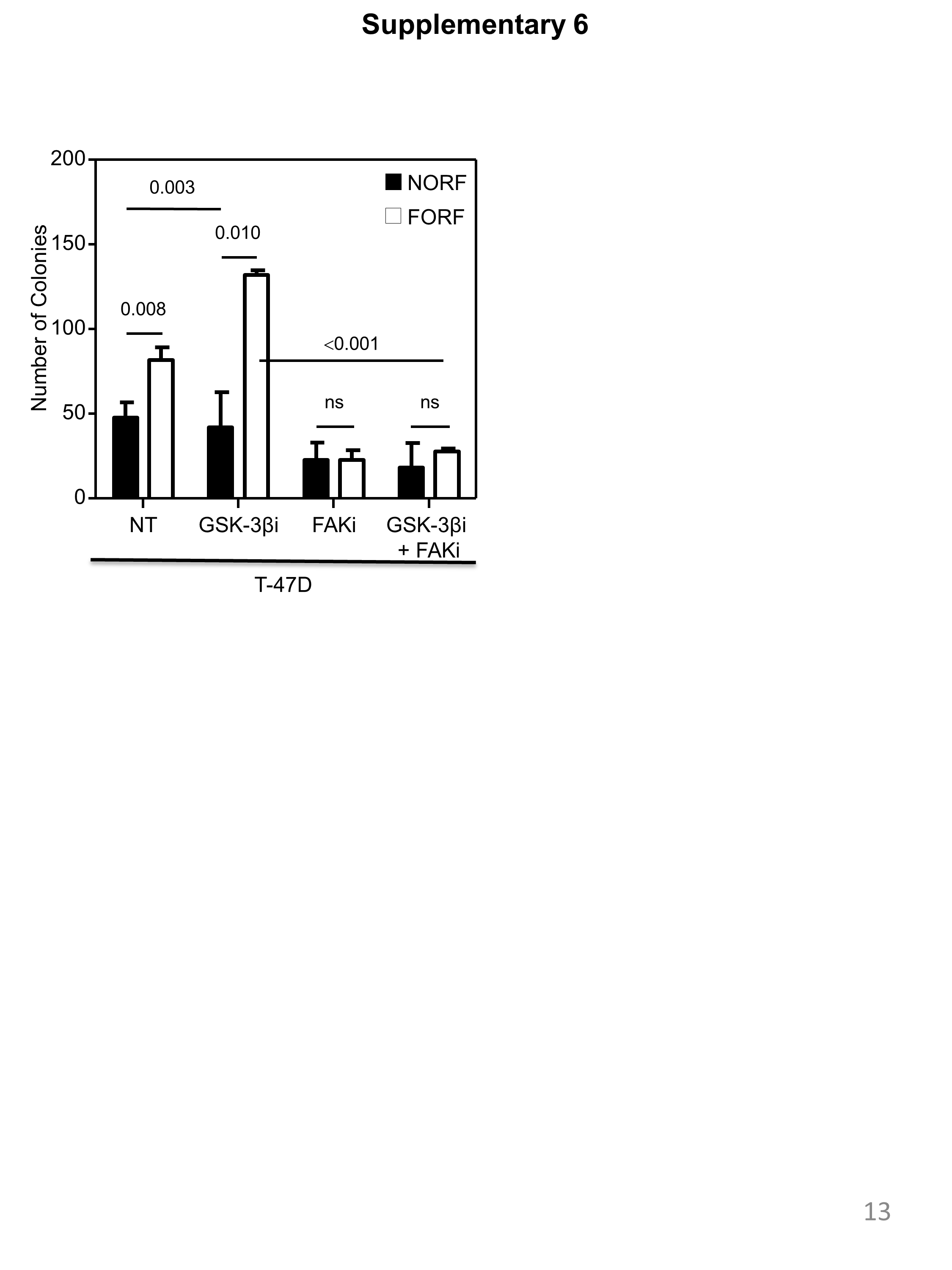

Supplement: Supplementary Figure 6 — Induction of fascin expression in the fascin-negative T-47D breast cancer cells increases their activation of β-catenin signaling pathway and enhances their colony formation ability in a FAK-dependent manner. Colony formation was assessed after fascin expression (FORF) in T-47D relative to NORF group in the presence or absence of GSK-3βi ± FAKi. Bar graph showing the number (mean of triplicates ± SD) of colonies of 3 independent experiments. [file Image_6.TIF]
